# Seasonal Influenza A (H1N1) Infection in Early Pregnancy and Second Trimester Fetal Demise

**DOI:** 10.3201/eid1701.091895

**Published:** 2011-01

**Authors:** Richard W. Lieberman, Natasha Bagdasarian, Dafydd Thomas, and, Cosmas Van De Ven

**Affiliations:** Author affiliation: University of Michigan Health System, Ann Arbor, Michigan, USA

**Keywords:** Viruses, influenza, seasonal influenza, transplacental passage, intrauterine fetal demise, IUFD, pregnancy, expedited, dispatch

## Abstract

A second trimester fetal demise followed influenza-like illness in early pregnancy. Influenza A virus (H1N1) was identified in maternal and fetal tissue, confirming transplacental passage. These findings suggested a causal relationship between early exposure and fetal demise. Management of future influenza outbreaks should include evaluation of products of conception associated with fetal loss.

Increased maternal illness and death appeared in early reports of influenza A pandemic (H1N1) 2009. Three pregnancy-related complications associated with pandemic (H1N1) 2009 were reported in May 2009: 1 postpartum maternal death, 1 preterm birth, and 1 early second trimester (weeks 14–20) loss (*1*). In April 2010, a summary of pandemic (H1N1) 2009 among 788 pregnant women demonstrated a disproportionately high risk for death; the rate of spontaneous miscarriage was 1.4%, but details were not provided (*2*). Correlating influenza exposure to pregnancy loss is not straightforward because first trimester miscarriage is common, second trimester loss before 24 weeks is not well studied (*3*), and viral identification in products of conception has rarely been attempted. We report a second trimester fetal demise that occurred after exposure to seasonal influenza A virus (H1N1) early in pregnancy.

## The Case

In 2008, a 30-year-old primigravida physician experienced intrauterine fetal demise (IUFD) at 20 weeks’ gestation, as established by last menstrual period and confirmed by ultrasound at 8 weeks. The patient spent most of her pregnancy providing direct patient care. In October 2007, during gestational weeks 2–6, she was exposed to at least 2 patients with presumed influenza. She reported symptoms of fever, myalgia, headache, and cough during gestational week 4. She had no testing for influenza, did not receive antiviral therapy, and took acetaminophen; her symptoms resolved. She received the killed seasonal influenza vaccine at gestational week 6. An ultrasound at 18 weeks demonstrated early growth restriction and oligohydramnios with normal appearing fetal kidneys and head. IUFD was noted at 20 weeks.

The patient had an unremarkable medical history, did not smoke, and was normotensive throughout pregnancy. Antibodies to cytomegalovirus and toxoplasmosis were not detected.

**Figure 1:**
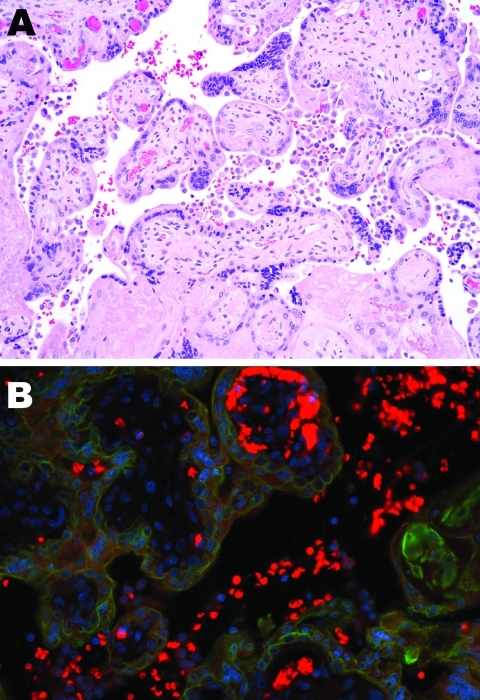
Tissue sample from 30-year-old primigravida patient exposed to seasonal influenza (H1N1). A) Intervillous (maternal) spaces with clusters/sheets of histiocytes (chronic intervillositis) and fibrotic fetal chorionic villi with Hofbauer cells–histiocytic inflammation (hematoxylin and eosin stain, original magnification ×200). B) Dual-stained immunofluorescent assay showing antibodies to influenza A virus (H1N1) (tetramethylrhodamine isothiocyanate, red) and cytokeratin (fluorescein isothiocyanate, green) in intravillous (fetal) and intervillous (maternal) space.

The immature placenta showed no visible abnormalities. Microscopically, histiocytes were abundant in the maternal space (chronic intervillositis) and were noted within the fetal chorionic villi (Hofbauer cells; Figure 1, panel A). Histologic analysis of fetal autopsy specimens showed scattered luminal histiocytes of the lung and gut. No other inflammatory changes or viral inclusions were identified in the placenta or fetal organs.

**Figure 2:**
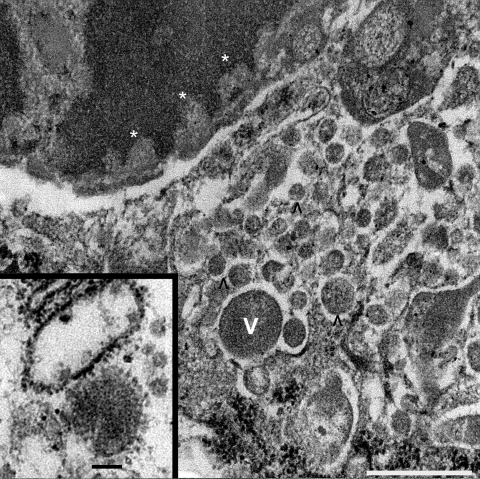
Tissue sample from 30-year-old primigravida patient exposed to seasonal influenza (H1N1). Electron microscopy (original magnification ×70,000) of maternal intervillous space and fetal chorionic villi, showing intranuclear viral transcription aligning along the nuclear envelope–electron hypodensities (asterisks), and intracytoplasmic viral production in varying stages shown by numerous electron densities (V and ^). Scale bar = 500 nm. Inset: enlarged image of mature virion with capsular projections. Ultrastructural features are characteristic of influenza viruses. Scale bar = 100 nm.

Chronic intervillositis was evident in the placental histology and has been previously described with nonspecific changes in miscarriage and recurrent pregnancy loss (*4*). Formalin-fixed placental tissue was imaged with electron microscopy. Histiocytes identified from the maternal intervillous space and fetal chorionic villi demonstrated characteristics of viral production (Figure 2). Several well-formed viral capsids were noted within the cytoplasm, each containing regularly spaced projections along the surface of the virion corresponding to the hemagglutinin and neuraminidase spikes.

PCR for M1 capsid protein confirmed influenza A. Immunohistochemical testing was performed on tissue from the placenta and on a sampling of fetal organs by using influenza A virus (H1N1)–specific antibody. In the placenta, stippled immunofluorescence (Figure 1, panel B) was noted in the histiocytes of the intervillous space (maternal) and fetal intravillous histiocytes. In the fetal organs, immunofluorescence for subtype H1N1 was notable in the surface epithelial cells and scattered luminal histiocytes of the fetal respiratory and gastrointestinal tracts. No immunofluorescence was noted within the fetal organs, which implied transamnionic passage of virus (amnionic fluid infection). The fetus was XY karyotype. Y-specific immunofluorescent stain with histiocyte counterstaining was performed. Because only the intravillous (fetal) histiocytes stained positively, maternal and fetal inflammatory responses were confirmed.

## Conclusions

Harris reported higher rates of miscarriage after exposure and infection in the earliest months of pregnancy in the Spanish influenza outbreak of 1918 (subtype H1N1) (*5*). Hardy et al. reported a similar observation following the Asian influenza outbreak of 1957–58 (*6*), as did investigators in the United Kingdom during the 1985–86 influenza season (*7*).

Several case reports document transplacental transfer of influenza virus. Yawn et al. documented influenza A transplacental transfer from the mother to amniotic fluid and fetal heart (*8*). Mel'nikova et al. summarized placental findings associated with influenza infections in publications from 1987 and 1994, describing perivillous and villous inflammation with concurrent amniotic fluid immunofluorescence for influenza virus (*9*). In another study (*10*), researchers from Beijing, People’s Republic of China, reported autopsy findings for 2 patients (1 of whom was 4 months pregnant) who died of avian influenza A (H5N1). Viral antigens were present in the maternal pulmonary system, and virus was detected in placental Hofbauer cells (histiocytes), cytotrophoblast, fetal lung, circulating mononuclear cells, and liver macrophages (*10*). Their documentation of tropism of the influenza virus to placental tissue is similar to the histopathologic features in our report. Our unexpected discovery of viral production in placental, maternal, and fetal histiocytes is another example of transplacental passage of influenza.

Miscarriage or completion of pregnancy may follow nonfatal maternal influenza infection. Adverse fetal outcomes have reportedly included congenital malformation and schizophrenia, but factors that determine these effects remain unclear (*11**,**12*). Fetal consequences of influenza exposure may be an effect of systemic inflammatory response to the infection, which could represent direct action of the virus on the placenta, the maternal–fetal interface, or the fetus. Uchide et al. reported the inflammatory response of decidual and fetal tissue in the presence of influenza virus infection and demonstrated expression of mRNA for a set of proinflammatory cytokines such as interleukin-6, tumor necrosis factor-α, and granulocyte–macrophage colony-stimulating factor, secreted in substantial amounts in response to exposure to influenza virus in vitro (*13*). As our case suggests, a complex interaction of maternal and fetal responses at the interface is a more likely explanation.

A total of 15%–20% of pregnancies end in spontaneous miscarriage, most before gestational week 12 (*3*). Approximately 50% of miscarriages are associated with chromosomal defects, which leaves many other miscarriages unexplained (*14*). Redline et al. examined first trimester miscarriages not associated with chromosomal abnormality, observing histologic changes of chronic intervillositis and increased perivillous fibrin (*15*). The mechanism of loss was thought to be related to a maternal inflammatory response to infectious antigen or an autoimmune phenomenon. Whether these findings can be extrapolated to second trimester losses is unknown. Second trimester miscarriages account for only 1%–2% of all pregnancy losses (*3*). Histopathologic features of these early second trimester miscarriages are not well studied, and descriptions are usually included in reports of fetal losses during 14–27 weeks, which include IUFD (>20 weeks) and miscarriage. Consequently, a direct causal relationship between influenza A and fetal demise has not been well established.

In our report, a retrospective investigation of the patient’s products of conception with ultrastructural, immunohistochemical, and molecular methods that identified subtype H1N1 implies an exposure during her first trimester. Type-specific serologic analysis was not possible because she received influenza vaccine several weeks after presumed exposure. The maternal and fetal inflammatory responses (chronic intervillositis) after placental infection may have reduced villous surface area for oxygen and nutrient transport, resulting in growth restriction and ultimately second trimester loss.

Investigation of the effects of influenza in early pregnancy is needed to evaluate potential pathophysiological relationships between maternal exposure, infection, and fetal loss or adverse outcome. During the 2009–10 influenza pandemic, the Centers for Disease Control and Prevention (Atlanta, GA, USA) collected information from populations exposed to and infected with pandemic (H1N1) 2009 virus. Products of conception were not included among the samples cataloged. We recommend that future influenza outbreak evaluations of women exposed to influenza with first or early second trimester pregnancy loss include collections of placental and fetal tissue samples.

## References

[1] Centers for Disease Control and Prevention. Novel influenza A (H1N1) virus infections in three pregnant women—United States, April–May 2009. [Erratum in MMWR Morb Mortal Wkly Rep. 2009;58:541]. MMWR Morb Mortal Wkly Rep. 2009;58:497–500. 19444154

[2] Siston AM, Rasmussen SA, Honein MA, Fry AM, Seib K, Callaghan WM, Pandemic 2009 influenza A(H1N1) virus illness among pregnant women in the United States. JAMA. 2010;303:1517–25. 10.1001/jama.2010.479 20407061 PMC5823273

[3] Goldenberg RL, Culhane JF, Johnson DC. Maternal infection and adverse fetal and neonatal outcomes. Clin Perinatol. 2005;32:523–59. 10.1016/j.clp.2005.04.006 16085019 PMC7119141

[4] Redline RW. Placental pathology: a systematic approach with clinical correlations. Placenta. 2008;29(Suppl A):S86–91. 10.1016/j.placenta.2007.09.003 17950457

[5] Harris JW. Influenza occurring in pregnant women: a statistical study of 1350 cases. J Am Med Assoc. 1919;72:970–2.

[6] Hardy JM, Azarowicz EN, Mannini A, Donald N, Medearis J. The effect of Asian influenza on the outcome of pregnancy, Baltimore, 1957–1958. Am J Public Health Nations Health. 1961;51:1182–8. 10.2105/AJPH.51.8.1182 13711529 PMC1522235

[7] Stanwell-Smith R, Parker AM, Chakraverty P, Soltanpoor N, Simpson CN. Possible association of influenza A with fetal loss: investigation of a cluster of spontaneous abortions and stillbirths. Commun Dis Rep CDR Rev. 1994;4:R28–32. 7513232

[8] Yawn DH, Pyeatte JC, Joseph JM, Eichler SL, Garcia-Bunuel R. Transplacental transfer of influenza virus. JAMA. 1971;216:1022–3. 10.1001/jama.216.6.1022 5108246

[9] Mel’nikova VF, Tsinzerling VA, Aksenov OA, Tsinzerling AV. Chronic course of influenza with extrapulmonary involvement [in Russian]. Arkh Patol. 1994;56:33–8. 8204049

[10] Gu J, Xie Z, Gao Z, Liu J, Korteweg C, Ye J, H5N1 infection of the respiratory tract and beyond: a molecular pathology study [comment]. Lancet. 2007;370:1137–45. 10.1016/S0140-6736(07)61515-3 17905166 PMC7159293

[11] Acs N, Banhidy F, Puho E, Czeizel AE. Maternal influenza during pregnancy and risk of congenital abnormalities in offspring. Birth Defects Res Part A Clin Mol Teratol. 2005;73:989–96. 10.1002/bdra.20195 16323157

[12] Selten JP, Frissen A, Lensvelt-Mulders G, Morgan VA, Selten J-P, Frissen A, Schizophrenia and 1957 pandemic of influenza: meta-analysis. Schizophr Bull. 2010;36:219–28. 10.1093/schbul/sbp147 19959706 PMC2833128

[13] Uchide N, Ohyama K, Bessho T, Toyoda H. Induction of pro-inflammatory cytokine gene expression and apoptosis in human chorion cells of fetal membranes by influenza virus infection: possible implications for maintenance and interruption of pregnancy during infection. Med Sci Monit. 2005;11:RA7–16. 15614205

[14] Goddijn M, Leschot NJ. Genetic aspects of miscarriage. Best Pract Res Clin Obstet Gynaecol. 2000;14:855–65. 10.1053/beog.2000.0124 11023805

[15] Redline RW, Zaragoza M, Hassold T. Prevalence of developmental and inflammatory lesions in nonmolar first-trimester spontaneous abortions. Hum Pathol. 1999;30:93–100. 10.1016/S0046-8177(99)90307-6 9923934

